# Primary jejunogastric intussusception: A case report and review of the literature

**DOI:** 10.1016/j.ijscr.2021.106666

**Published:** 2021-12-07

**Authors:** Giovambattista Caruso, Chiara Toscano, Mariapia Gangemi, Giuseppe Evola, Carlo Reina, Giuseppe Angelo Reina

**Affiliations:** a General Surgery Department, Santissimo Salvatore Hospital (ASP Catania), Paternò, Catania, Italy; b Thoracic Surgery Department, Policlinico–San Marco Hospital, Catania, Italy; c General and Emergency Surgery Department, Garibaldi Hospital, Catania, Italy

**Keywords:** Jejunogastric intussusception, COVID 19, Hemorrhagic infarction, Case report

## Abstract

**Introduction:**

Jejunogastric intussusception following gastric surgery is a rare complication that, if not diagnosed early, can have catastrophic outcomes.

**Presentation of case:**

We have reported a case, never described previously, of an acute spontaneous retrograde JGI, presenting with obstruction and hematemesis, in a 70-year-old woman who has never, previously, undergone abdominal surgery.

**Discussion:**

As in all cases of intestinal intussusception, early diagnosis is important for acute JGI as mortality rates increase from 10% when the intervention occurs within 48 h. to 50% if treatment is delayed for 96 h.

The diagnosis of JGI can be determined with many imaging studies, such as endoscopy, ultrasonography (US), barium stadium and CT scan.

Although JGI, up to now, has been described as a rare complication after any type of gastric surgery, this disease must, however, be suspected also in patients who have never undergone abdominal surgery, if they present with non-sedable abdominal pain associated with signs of high intestinal obstruction and hematemesis.

**Conclusion:**

Our hope is to add to the available literature to aid physicians in their diagnostic work-up and in developing management plans for similar cases occurring in the future.

## Highlights

• Jejunogastric intussusception following gastric surgery is a rare complication.
• Primary jejunogastric intussusception in patient who never underwent previous abdominal surgery had never been described.
• Early diagnosis of this condition is crucial for early surgical intervention.
• Immediate treatment is critical to avoid catastrophic outcomes.

## 1 Introduction

Jejunogastric intussusception (JGI) is a rare complication of gastric surgery, especially of gastrectomy or gastrojejunostomy, which can occur any time after the gastric operation [1].

Early diagnosis of this condition is crucial for early surgical intervention. In the literature, the incidence is less than 0,1% of all gastric resection [2]. When the operation is performed within 48 h, the mortality rate is about 10%. By contrast, operation delayed beyond 48 h may be associated with a mortality rate of up to 50% [3]. The classic triad of acute JGI, present in only 50% of patients, includes epigastric pain, vomiting with or without hematemesis, and a palpable epigastric mass [4].

Emergency surgery is the treatment of choice for acute JGI. Traditionally, laparotomy or laparoscopy for reduction or resection of the intussusception is the main surgical method [5].

Here, we present the first case of spontaneous jejunogastric intussusception in a 70-year-old woman who never underwent previous abdominal surgery.

The present work has been reported in accordance with the Surgical Case Reports (SCARE 2020) criteria [6].

## 2 Case report

In February 2021, a 70-year-old woman presented to emergency department with a 5-day history of non-sedable diffuse epigastric pain. He was associated with vomiting, initially bilious and then hematemesis, fever, paroxysms of cough and dyspnea. No history of previous abdominal surgery in the past.

Heavy smoker patient. In anamnesis: chronic obstructive bronchopathy, chronic atrial fibrillation, arterial hypertension, a primary hyperparathyroidism from known parathyroid adenoma and an inveterate umbilical hernia.

The patient reported, for about 30 years, repeated episodes of epigastric pain, with alimentary vomiting, which resolved spontaneously a few hours after onset, attributed to episodes of incarceration of the umbilical hernia.

At the admission in the emergency room: Blood pressure: 105/55 mm Hg; heart rate: 102/min; external body temperature: 38,2 °C; sPO2: 91% in ambient air.

Physical examination of the abdomen revealed a voluminous umbilical hernia, not reducible, particularly painful on palpation. For the rest, the abdomen was flat, not breathing, hardly treatable and showed signs of defensive contracture and peritonism.

Blood tests showed a hemoconcentration with HT: 50.5%, WBC: 10,600/mm^3^; hyperamylasemia (2768 IU/l) and hyperlipasemia (1577 IU/l), γGT: 63 IU/l; AST: 75 IU/l, creatinine: 1.20 mg/dl; CRP: 45 mg/l.

Arterial blood gas analysis: pO2: 42.4 mm Hg; pH: 7.48; pCO2: 51.7 mm Hg; HCO3: 39 mmol/l; K: 3.3 mEq/l, Na: 152 mEq/l.

The indirect intra-abdominal pressure was 6 mm Hg but, under coughing, it reached values of 25 mm Hg and more.

Real time polymerase chain reaction (RT-PCR) assay to detect SARS-CoV-2 RNA was positive.

CT examination of the chest and abdomen diagnosed necrotic-exudative pancreatitis with peripancreatic fluid collections. Intrapancreatic choledochus diameter: 11 mm.

**Fig. 1:**
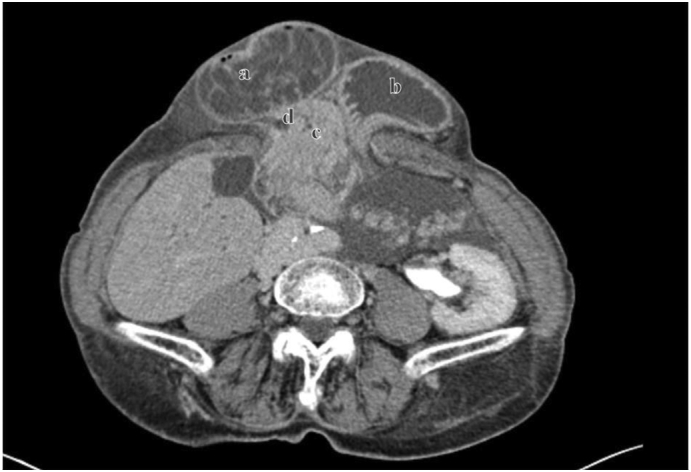
CT images. a: Duodenum; b: stomach; c: head of pancreas; d: choledochus.

**Fig. 2:**
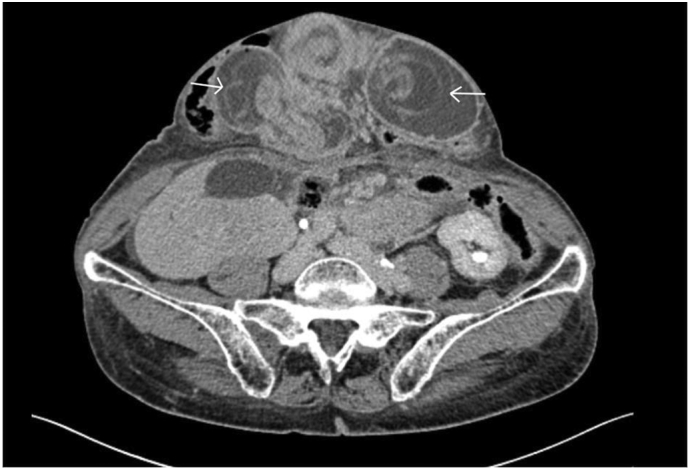
CT Images. Arrows indicate intussusception or “target sign”.

In the umbilical area, a large hernia with a 50 mm diameter hernial port and engagement of the intestinal loops, the proximal duodenum, the distal stomach and the head of the pancreas (Fig. 1). Retrograde invagination of the jejunum from Treitz to the stomach was also described with signs of distress of the intestinal wall and complete intestinal obstruction (Fig. 2).

Multiple ground glass foci in both lungs.

Once the gastric-nose tube was placed, approximately 250 cm^3^ of hemorrhagic fluid were aspirated.

**Fig. 3:**
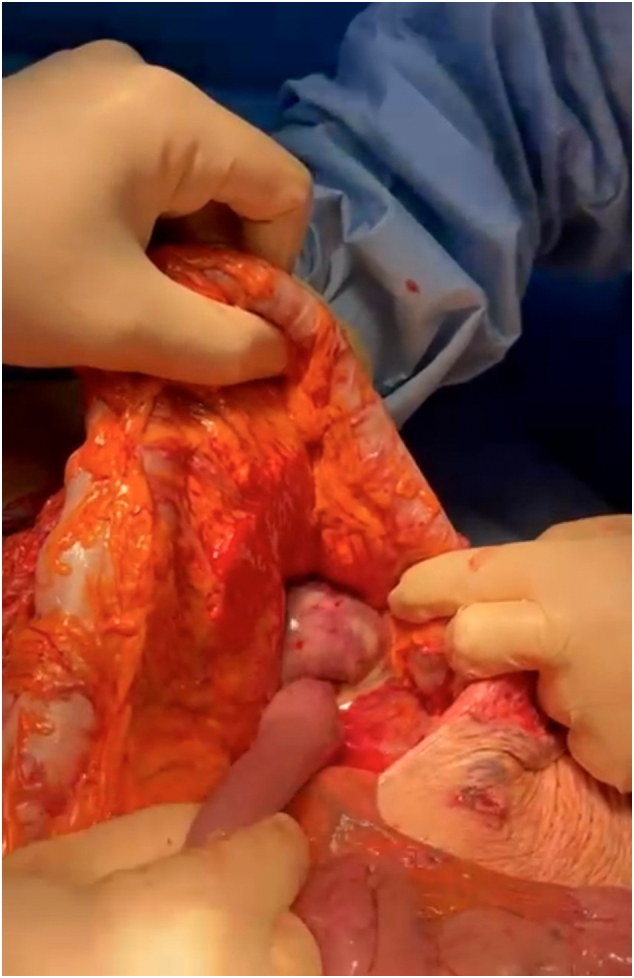
Intraoperative image. Jejunum invaginates in the Treitz.

**Fig. 4:**
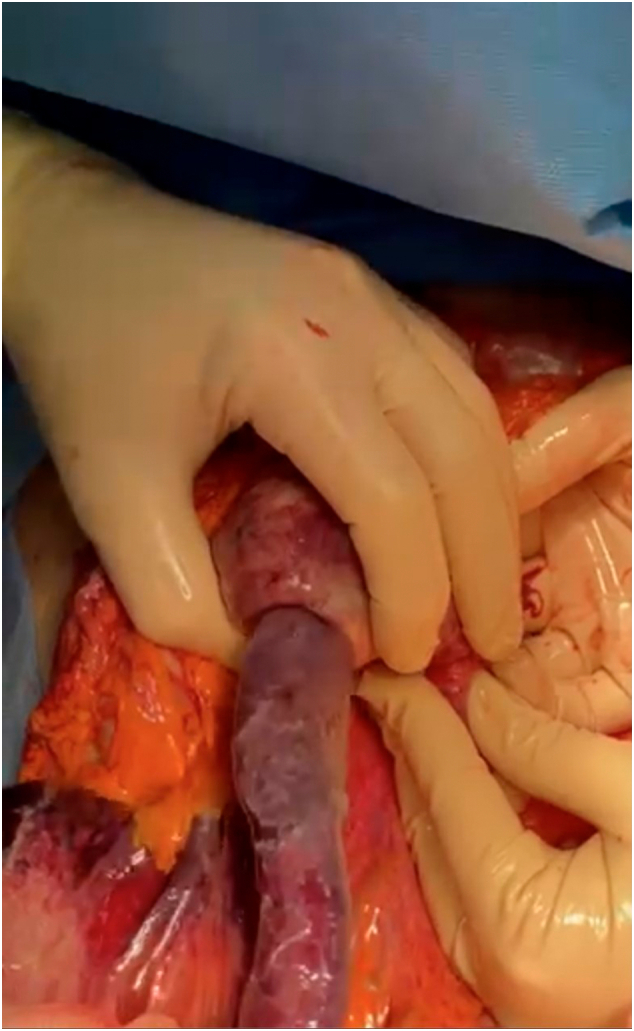
Intraoperative image. Manual reduction of intussusception.

**Fig. 5:**
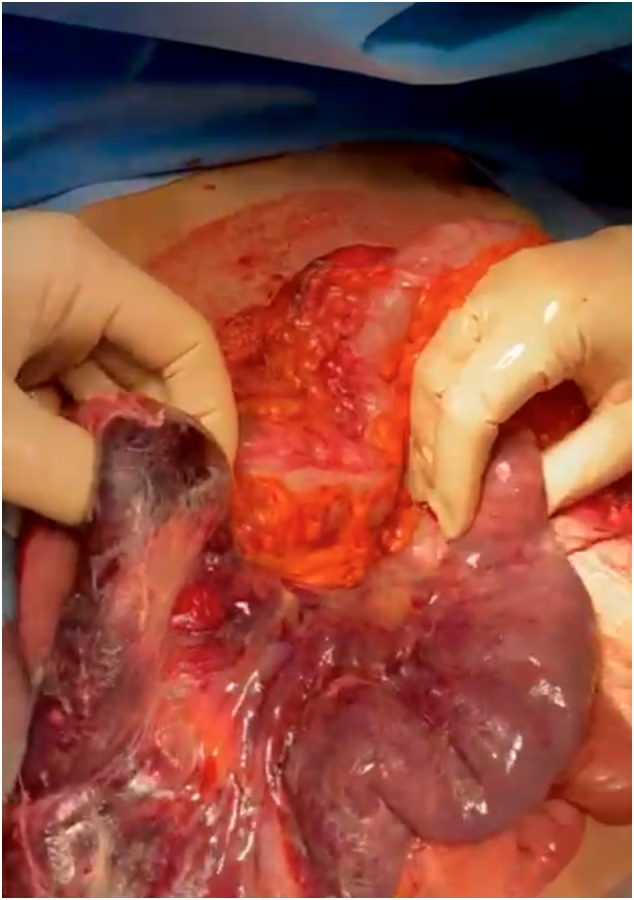
Intraoperative image. Jejunum invaginated in hemorrhagic infarction.

After surgical evaluation and informed consent was obtained, the patient was immediately transferred to the operating room where she underwent an emergency laparotomy. After the lysis of viscero-parietal adhesions and the reduction of the hernial contents in the abdomen, a significant distension of the duodenum and gastric antrum from retrograde invagination of the jejunum from the Treitz to the stomach was highlighted in abdomen. Therefore, manual reduction of the invaginated intestine was carried out with the release of about 60 cm of intestine which showed significant signs of hemorrhagic infarction and multiple diastatic perforations (Fig. 3, Fig. 4, Fig. 5).

A prompt resection of the intestinal piece was performed with jejuno-jejunal stapled side-to-side anastomosis.

The surgery was completed with simple hernioplasty and placement of two abdominal drains.

The surgery lasted 75 min.

Blood losses were quantified as less than 50 cm^3^.

Transferred to the Resuscitation Unit, the post-operative course was characterized by progressive and constant worsening of respiratory exchanges (P_a_O_2_: 34 mm Hg; P_a_CO_2_: 60 mm Hg; P_a_O_2_/FiO_2_ < 100 mm Hg), linked to SARS-COV2 pneumonia, which unfortunately complicated the severe septic situation, leading to death the patient in the third postoperative day for severe ARDS.

## 3 Discussion

Adult intussusception is rarely observed compared with that in children, accounting for 5% of intussusception and ∼0.003% to 0.02% of all hospital admissions, less than 10% of these cases affect the gastro-duodenal region.

Patients with this condition often have nonspecific symptoms, typically characterized by epigastric pain and vomiting.

If untreated, intussusception can cause ischemia of the invaginated bowel wall and consequent perforation with peritonitis [7], [8].

Jejunogastric intussusception is a rare complication after gastric surgery.

It was first described in 1914 by Bozzi, 30 years after the first gastrojejunostomy was performed [9].

To date, only 300 cases of jejunogastric intussusception have been reported worldwide and all after previous gastric surgery [10].

It is classified into 4 types:

- Type 1: afferent loop intussusception.
- Type 2: efferent loop intussusception.
- Type 3: combined afferent and efferent loop intussusception.
- Type 4: intussusception through side-to-side jejunal anastomosis (Braun) [11].

The cause(s) of the jejunogastric intussusception is (are) poorly understood. Various factors have been incriminated such as hyperacidity long afferent loop, jejunal spasm with abnormal motility, increased intra-abdominal pressure, retrograde peristalsis, etc. Probably, retrograde peristalsis, which can occur in normal people prior to gastric surgery, seems to be accepted as the cause of type 2 jejunogastric intussusception by most authors [12].

Two forms of JGI have been clinically recognized: an acute and a chronic form. In the acute form, incarceration and strangulation of the intussuscepted loop generally occur whilst spontaneous reduction is usual in the chronic type. Thus, the acute form is characterized by acute severe colicky epigastric pain, vomiting and, subsequently, hematemesis. Epigastric tenderness and a palpable abdominal mass can be observed in about 50% and signs of high intestinal obstruction can also be found [1]. These symptoms are the classic triad of JGI in a patient with a previous gastric surgery [13].

Early diagnosis is important for acute JGI as mortality rates increase from 10% when the intervention occurs within 48 h. to 50% if treatment is delayed for 96 h [5], [11].

The diagnosis of JGI can be determined with many imaging studies, such as endoscopy, ultrasonography (US), barium stadium and CT scan.

Endoscopy is the diagnostic procedure for the patient with hematemesis, but the intussusception could be mistaken as an immobile clot or a bezoar. US findings of intussusception classically reveal a mass with echogenic center surrounded by concentric echogenic rings with a peripheral rim of hypo echogenicity, described as “pseudo kidney” sign.

The typical CT finding of intussusception is a soft tissue mass with a “sausage” or “target” appearance [13].

The treatment of JGI consists mainly of surgery using several approaches depending on the intraoperative findings:

- Manual reduction of the intussusception
- Revision of the anastomosis
- Anchoring of the efferent limb to the parietal peritoneum or suturing together of the afferent and efferent limbs after reduction of the intussusception
- New gastrojejunostomy using a Roux-en-Y reconstruction.

If the intussusception is gangrenous, resection and revision of anastomosis provide the correct treatment [12].

Endoscopic reduction has been reported in some case reports. However, endoscopic reduction of JGI is contraindicated when the peritoneal signs are suspected.

Furthermore, endoscopic reduction of JGI has a significant risk of recurrence [5].

We reported a case, never described previously, of an acute spontaneous retrograde JGI, presenting with obstruction and hematemesis, in a 70-year-old woman never underwent to previous abdominal surgery. In this case, probably, the inveterate voluminous umbilical hernia with herniation of the stomach, duodenum and head of the pancreas, the increase in abdominal pressure under repeated coughing, acute pancreatitis and abdominal effusion, were the factors triggering a retrograde peristalsis with consequent invagination.

The histological examination of the surgical piece did not reveal any neoplasms or lesions that could have acted as a lead point.

Although the diagnosis was made immediately and the patient underwent emergency surgery, her delayed access to the emergency room, associated with concomitant severe acute pancreatitis and SARS-COV 2 pneumonia, was responsible for the unfortunate outcome.

JGI is a rare but potentially fatal disease.

For this reason, early diagnosis and surgical treatment are crucial.

## 4 Conclusion

This is a very rare case of gastric jejunum intussusception, never reported in the medical literature. Our hope is to add to the available literature in order to help physicians in their diagnostic work-up and in developing management plans for similar cases occurring in the future.

Although JGI, up to now, has been described as a rare complication after any type of gastric surgery, this disease must, however, be suspected also in patients who have never undergone abdominal surgery, if they present with non-sedable abdominal pain associated with signs of high intestinal obstruction and hematemesis.

Immediate treatment is critical to avoid catastrophic outcomes, and, therefore, a high index of suspicion is required for early diagnosis.

## Consent

Written informed consent was obtained from the patient for publication of this case report and accompanying images.

## Provenance and peer review

Not commissioned, externally peer-reviewed.

## Ethical approval

Ethic approval has been exempted by our institution because this is a case report and no new studies or new techniques were carried out.

## Funding

This research did not receive any specific grant from funding-agencies in the public, commercial, or non-for-profit sectors.

## Guarantor

The guarantor for this case report is Giovambattista Caruso.

## Research registration number

This case report does not require registration as a research study.

## CRediT authorship contribution statement

Giovambattista Caruso: conceptualization, writing original draft, review and editing, data curation.
Chiara Toscano: Resources, data curation.
Mariapia Gangemi: Conceptualization, software and video editing.
Giuseppe Evola: Review and editing.
Carlo Reina: Writing, review and editing.
Giuseppe Reina: Supervision.

## Declaration of competing interest

All the authors certify that there is no conflict of interest regarding the material discussed in the manuscript.

## Appendix A Supplementary data

Supplementary video https://vimeo.com/637230421. Supplementary video
